# Case of Ovarian Dropsy, Treated by Tapping and Iodine Injection

**Published:** 1846-06

**Authors:** Benjamin A. Allison

**Affiliations:** Spencer, Indiana


*Case of Ovarian Dropsy, treated by Tapping and Iodine Injection.* By Benjamin A. Allison, M. D., of Spencer, Indiana.

On Nov. 30th, 1844, I was called to consult with Dr. F——, on the propriety of tapping Mrs. C——, a countrywoman residing thirteen miles from this place, whom I found in the following situation : Countenance dusky, haggard and anxious, pulse feeble, thready and irregular, nausea and vomiting, great distention and tenderness of abdomen, destressing dyspnœa. She had had no evacuation of urine or feces for forty-eight hours, and no sleep for that length of time. The medical treatment she had received was solely with the view of evacuating her bowels; she had been bled, taken salts and senna, cathartic pills, castor and croton oils, injections, &c. Under existing circumstances, I introduced the catheter, when a quantity of scalding, offensive urine came away, affording slight relief. Then gave her a full opiate, and ordered her bowels to be fomented. She rested comparatively easy during the night, had a refreshing nap, and awoke on the following morning considerably relieved. Upon a pretty full examination of the patient and the account given by her husband, I learned the history of the case to be as follows: Age 35 years, mother of two children, and now five months pregnant with the third. Was remarkably healthy until 21 years of age—the date of her marriage. Two weeks after the nuptials, "the custom of women being upon her," she was exposed to a cold chilly rain, and suppression of the discharge followed.

About a month from this time she noticed a tumor in the pubic region; she thinks it was rather to the right of the median line; was the size of a hen's egg, tender to the touch, and throbbed when pressed upon. In due time her menses became re-established, but attended with pain in the uterus, headache, and loss of appetite.

The tumour gradually increased until about the fourth year, at which time it was larger than a man's head, and could be moved from one side of the abdomen to the other. During these four years she had three abortions. The tumor now became stationary, and in process of time she discovered herself pregnant, went her full time and gave birth to a healthy infant.

In little upwards of a year after the birth of this child, she gave birth to a second. After the birth of the latter the tumour began to increase sensibly, and by its weight and pressure soon made serious inroads on her general health.

She has suffered, from time to time, a variety of unpleasant symptoms, among which costiveness has been the most troublesome—giving rise, if not relieved, to sick-headache, great pain and swelling of the bowels.

She has been under the care of different physicians, some of whom pronounced the disease to be dropsy, whilst others regarded it as arising from the presence of worms in the bowels, and treated her accordingly.

Upon examination of her abdomen, fluctuation was evident. There was no question as to the presence of a fluid. Was it proper to tap, was the only question. She earnestly desired to be tapped; to use her own words, she "thought it might keep her from smothering to death." I operated, and owing to her pregnant state, introduced the trocar considerably higher up than is usual. I drew off gradually upwards of four gallons of water, and left about as much in her. After she rallied from the effects of the operation she felt great relief, and on the following night her bowels responded to a mild cathartic. From this time the case was placed under my care. It would be useless to give you all the particulars of the case from that time to the present.

She enjoyed passable health until a few weeks before the birth of her child, which she carried nine months, but owing to her feeble situation did not suckle it. After this the dropsical effusion began to increase rapidly, and I soon found it necessary to tap her the second time; and so on, as occasion required, until she was operated on five times. The fluid was variously coloured; at first it was a sort of straw colour, then the colour of ley, then milky, with particles like wheat bran floating in it.

The remedial agent from which she appeared to derive most benefit was the iodide of potassium, in connection with squills and juniper berries. These produced a profuse discharge of urine without deranging her stomach and bowels. When such derangement did happen, whether from medicines or other causes, the pulv. Ip. com. generally gave decided relief. At times it appeared as though she would get well, but the relief was not permanent.

Her strength gradually failed, progressive emaciation followed, her extremities dwindled away to the least possible size, and following this, they swelled to such a degree that bandages were necessary to keep the integuments from giving way. Such was her condition when I tapped her the last time, August 9th, 1845. After I operated this time I concluded to introduce a tent, and thereby allow the fluid to escape as fast as it might accumulate. Accordingly I introduced a silk chord one sixth of an inch in diameter, and instructed her husband in its use. It had the desired effect; he drew off the fluid at his pleasure without causing any material pain.

Thus matters progressed until the last of November, at which time I visited her, and found that during my absence the disease had been gradually getting worse. The discharge had become less, but almost puriform, hectic had set in, and night sweats tormented her most grievously. She was a living skeleton, and utterly unable to turn herself in bed. I looked upon her condition as entirely hopeless, but concluded to inject a solution of iodine into the dropsical sack as a last resort. I did so, and the symptoms that followed were truly alarming, and could not be entirely controlled. They subsided, however, in a few days, and she commenced improving. The discharge rapidly decreased until it almost ceased entirely. The superficial veins became visible, and her sallow features were lighted up with the glow of returning health. But her bowels were irregular, digestion feeble, and she suffered much with flatulence. To remedy this condition I put her upon a pill composed of aloes, quinine, sulph. iron, ginger, and ext. gentian. It had a very fine effect; kept her bowels regular, improved the tone of her stomach, and prevented flatulence. She continued the use of the pill for nearly four months.

A short time since her husband called to tell me her condition. She is enjoying better health than she has done for twelve years. Weighs about one hundred and fifty pounds, and is able to do her housework. Her colour is fresh and good.

The tent remains in, but the orifice is greatly inclined to heal; so much so, that processes of skin are thrown out around it. Occasionally the discharge amounts to a tea-spoon full of purulent matter in the course of twenty-four hours, and now and then it ceases altogether for a few days. She suffers considerably, especially of nights, with pains in her joints; they are also inclined to be stiff. She has had no return of the menses, but for four months past, at regular periods four weeks apart, she has had a discharge from the tent orifice, resembling the catamenial discharge in every particular; it continues five or six days, and discharges about a pint. She does not experience aphrodisiacal desires; the only feeling she has had that approached any thing of the kind, occurred one night in a dream.

[We have given the preceding case in the plain unvarnished language of the author, who is a young but respectable practitioner in the State of Indiana, in whose report we have entire confidence. The injection of iodine into the ovarian sac was certainly a hazardous experiment, to which he was doubtless led by the apparent hopelessness of the case, and the success which has attended its employment in dropsies of other serous cavities. The remedy is scarcely more dangerous, however, than ovariotomy, and in this instance seems to have been quite as fortunate. Nevertheless, we should ourselves hesitate before employing it.—Editor.]

